# Ciliary body length revisited by anterior segment optical coherence tomography: implications for safe access to the pars plana for intravitreal injections

**DOI:** 10.1007/s00417-020-04967-3

**Published:** 2020-10-19

**Authors:** Joel-Benjamin Lincke, Salome Keller, Joao Amaral, Martin S. Zinkernagel, Kaspar Schuerch

**Affiliations:** Department of Ophthalmology, Inselspital, University Hospital of Bern, Bern, Switzerland

**Keywords:** Ciliary body, Axial length, Anterior segment optical coherence tomography, Pars plana, Intravitreal injection

## Abstract

**Purpose:**

To investigate the dependence of the ciliary body length (CBL) on the axial length (AL) and to draw conclusions on implications regarding safe pars plana access for intravitreal injections and vitreoretinal surgery.

**Methods:**

A total of 200 individuals (mean age 42 years, SD ± 15.4) were enrolled in the study. Objective refraction and AL were obtained. Spherical equivalent (SE) was calculated. Anterior segment optical coherence tomography (ASOCT) was used to image and measure the CBL.

**Results:**

The mean SE was − 1.64 diopters (SD ± 3.15, range − 14.5 to + 9 diopters) and the mean AL was 24.19 mm (SD ± 1.65, range 19.8–32.2 mm). There was a significant correlation between SE and AL (*r*^2^ = 0.62, *p* < 0.0001). Mean CBL correlated significantly with age (*r*^2^ = 0.11, *p* < 0.0001), AL (*r*^2^ = 0.23, *p* < 0.0001) and SE (*r*^2^ = 0.25, *p* < 0.0001). The mean CBL was 3351 μm (SD ± 459, range 2184–4451 μm). Three separate groups were defined by their AL with a normal AL group (AL 22.5 to 25 mm), a short AL group (AL < 22.5 mm) and a long AL group (AL > 25 mm). The mean CBL in the normal AL group was 3311 μm (SD ± 427), in the short AL group 2936 μm (SD ± 335) and in the long AL group 3715 μm (SD ± 365), and differed significantly (*p* < 0.0001) when compared.

**Conclusion:**

For interventions requiring pars plana access (as an intravitreal injection or vitreoretinal surgery), an incision distance of 3.5–4.0 mm posterior to the limbus is recommended. In our research, however, a difference of 0.77 mm in mean CBL between the group with short AL and the group with long AL is demonstrated, implying that the mean CBL in very short and very long eyes differs significantly. These findings suggest that the AL should be taken into account for pars plana access and that it would be advisable to prefer the shorter or longer recommended distance (3.5 and 4.0 mm, respectively) from the limbus, which correlates with the AL. If AL is > 25 mm, a distance of 4.0 mm from the limbus should be chosen; and if AL is < 22.5 mm, a distance of 3.5 mm seems adequate.

**Trial registration number and date:**

NCT00564291, 27 Nov 2007
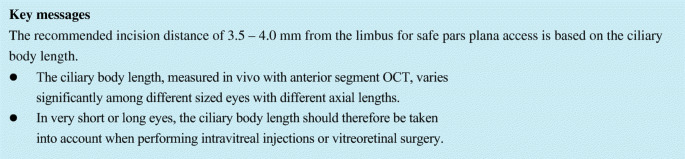

## Introduction

The ciliary body (CB) is located posterior to the iris [1]. It is composed of two parts, the pars plicata and the pars plana. The pars plicata forms the anterior portion and is contiguous to the posterior surface of the iris. It represents approximately one quarter of the whole CB. The pars plana, the posterior portion of the CB, is contiguous to the choroid at the ora serrata [2].

Intravitreal injections are applied through the pars plana of the CB, 3.5–4.0 mm posterior to the limbus [3]. For children, a distance of 0.5–3.5 mm from the limbus depending on the age is used [4, 5]. For this reason, it is important to have a thorough anatomical understanding of the CB and its extent.

One possible imaging modality to visualise these structures is optical coherence tomography (OCT). OCT is a non-invasive imaging modality obtaining high-resolution cross-sectional images of anatomical structures of the eye [6]. In 1994, OCT was first used to image structures of the anterior segment of the eye [7]. Until then, ultrasound biomicroscopy (UBM) was the standard method to visualise anterior segment structures [7]. Anterior segment optical coherence tomography (ASOCT) has several advantages. It is a non-invasive, non-contact technique and obtains a higher depth resolution compared with UBM [8, 9]. Previous studies assessed data of the CB using UBM [10, 11]. The ciliary body length (CBL) showed a significant positive correlation with the axial length (AL). Furthermore, CBL and ciliary body thickness (CBT) were found to be significantly increased in the superior quadrant compared with the nasal, temporal and inferior quadrants [12]. AL correlates with myopia: the higher the grade of myopia the longer the ocular bulb [13]. To analyse the CB in patients with a wide range of refractive errors, especially in myopic eyes, volume scans were performed in 4 quadrants using ASOCT. The objective of this study is to evaluate the total CBL (pars plicata + pars plana) using ASOCT in patients with differing refractive error to fathom the implications for interventions needing access to the pars plana of the CB, such as intravitreal injections and vitreoretinal surgery.

## Participants and methods

This study was conducted at the Department of Ophthalmology, University Hospital of Bern, Bern, Switzerland. All procedures used in this study were approved by the local Ethics Committee of the University of Bern, Switzerland, and adhered to the tenets of the Declaration of Helsinki. Over a period of 4 months (from November 2017 to February 2018), 200 participants were recruited for this prospective non-interventional study. From each individual, written informed consent was obtained. Male and female participants and patients aged > 18 years were acquired. Eyes were excluded if they had intravitreal injections, history of vitrectomy, pathologies of the sclera or macular pathologies. Pseudophakic eyes (*n* = 8) were not excluded, as they did not have surgery affecting the sclera. In each individual, primarily the right eye was included. If the right eye had to be excluded, the left eye was included. Objective refraction was obtained using Nidek ARK 1-s (Nidek CO. LTD, Aichi, Japan). The spherical equivalent was calculated and used for further analysis. A spectral domain ASOCT (Heidelberg Engineering GmbH, Heidelberg, Germany) was used to image the CB and the anterior sclera including the limbus. The ambient illumination was reduced to a minimum to have the fewest interfering signal. To obtain images of the anterior segment in 4 quadrants, all participants were asked to look at a fixation light that was moved from inferotemporal (IT) to superotemporal (ST), inferonasal (IN) and superonasal (SN).

To image a broad segment of the anterior structures of the eye, the scanned area with a pattern size of 15° × 5° (8.3 × 2.8 mm) was partially placed over the limbus. Each scanned area included 11 B-scans, which were separated by 277 μm from each other. Automatic real-time (ART) function was set at 36 frames to achieve better resolution.

**Fig. 1:**
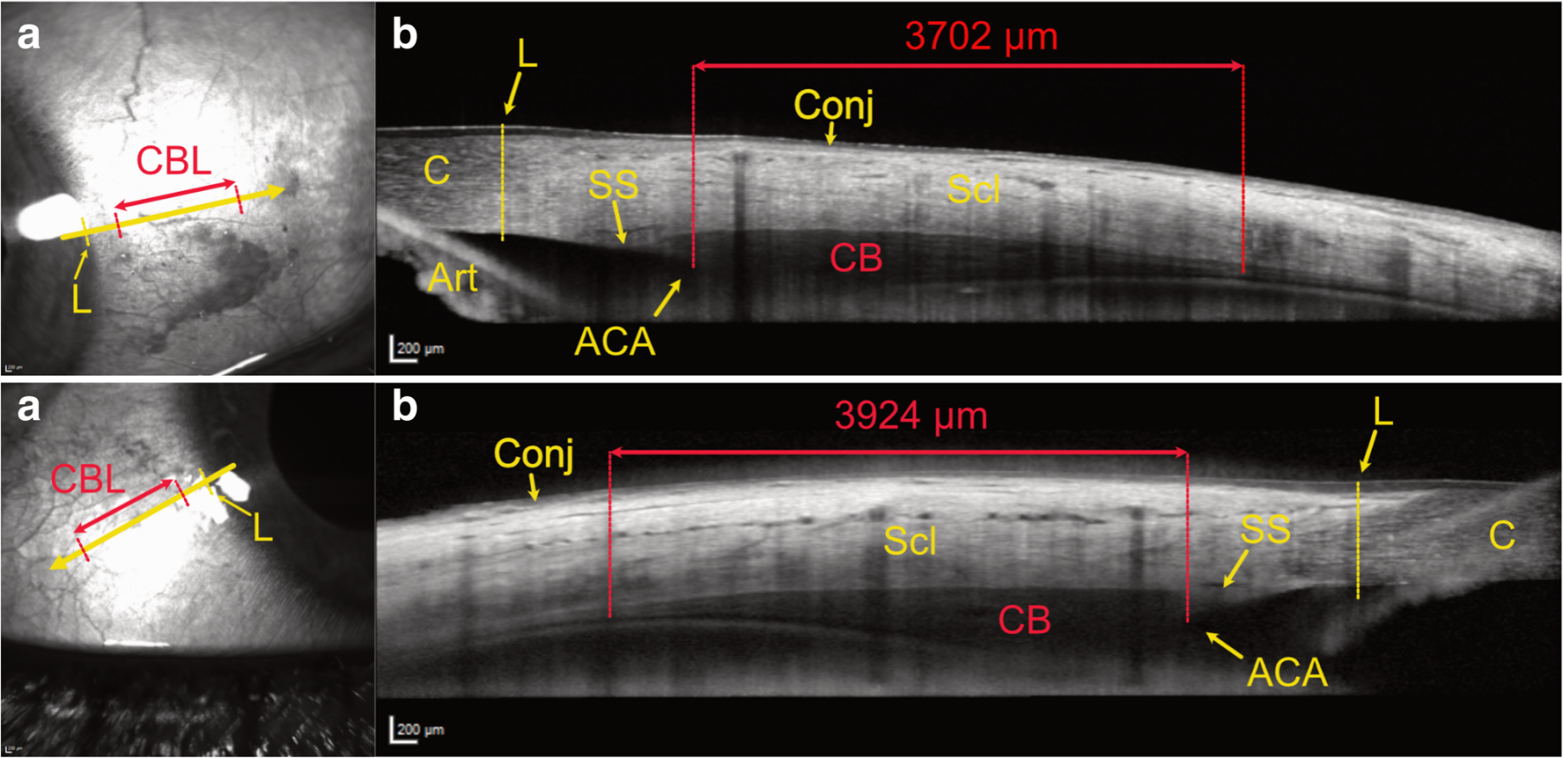
Two representative ASOCT images with infrared superficial en face images of the conjunctiva and underlying sclera near the limbus on the left (A) with the arrow indicating the cross section of the anterior segment optical coherence tomography (ASOCT) image on the right. The limbus (L) and the size of the ciliary body (CBL) are marked as measured on image (B), which illustrates the measured length of the ciliary body (in red), the ciliary body (CB) itself and the anterior chamber angle (ACA). L, limbus; CBL, ciliary body length; C, cornea; Scl, sclera; Conj, conjunctiva; SS, scleral spur; Art, artefact (inverted iris)

CBL was measured using the Heidelberg software (Heidelberg Eye Explorer Heyex 2, version 6.5.5.0, Heidelberg Engineering, Heidelberg, Germany). Measurements of the CBL were performed by two independent examiners (K.S. and A.J.) in 3 different B-scans for each quadrant after which the measurements were averaged. The mean calculated intraclass correlation coefficient (ICC) between the individual measurements was 0.91 (95% confidence interval, 0.89–0.93). The ciliary body length was measured as the distance in a straight line from the deepest point of the iridotrabecular angle to the last discernible mass of the ciliary body. The last discernible mass was defined as the region where there was no gap between the ciliary epithelium and the sclera and, after this point, the ciliary body epithelium or internal limiting membrane/retina continued parallel to the sclera (see Table 1 for an overview of the values and Fig. 1 for a representative illustration). IOL Master 500 (Carl Zeiss Meditec, Inc., Dublin, CA, USA, software version 7.7.4.0326) was used to measure AL. Five separate measurements of AL were averaged.

**Table 1:** Clinical characteristics of the enrolled subjects including ciliary body length (CBL) measurements with standard deviation (SD) and range

	Mean ± SD	Range
Age (years)	42.3 ± 15.4	19.1–79.6
Height (cm)	172.5 ± 8.9	148–200
Spherical equivalent (dpt)	− 1.64 ± 3.15	− 14.5–9.0
Axial length (mm)	24.19 ± 1.65	19.8–32.16
CBL mean (μm)	3351 ± 458.9	2184–4451
CBL superotemporal (μm)	3462 ± 497	2007–4734
CBL superonasal (μm)	3369 ± 460.5	2154–4716
CBL inferotemporal (μm)	3314 ± 539.6	1899–4829
CBL inferonasal (μm)	3238 ± 506	1836–4615

For statistical analysis, Prism 8 (GraphPad Software, Inc., La Jolla, CA, USA) was used. Data was analysed for normality using the d’Agostino-Pearson omnibus K2 test. AL and SE did not show a Gaussian distribution. Correlations including these parameters were done using Spearman’s rank correlation. For comparison of groups, ANOVA was used after verifying normality. A *p* value of 0.05 or smaller was considered statistically significant.

## Results

Of the 200 eyes enrolled in the study, 104 individuals were females and 96 males. The eyes in which one or multiple quadrants of the ciliary body could not be measured because important landmarks were not discernible or cut off were excluded. A total of 21 eyes were excluded, with 179 eyes left for CBL analysis.

**Fig. 2:**
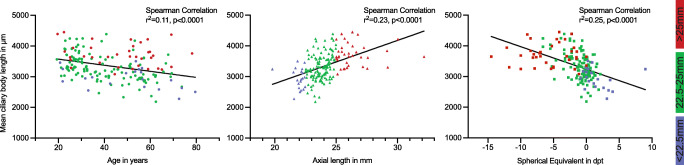
Correlations of the mean ciliary body length (CBL) with age, axial length (AL) and spherical equivalent (SE) from left to right, which showed a significant linear correlation. The *Y*-axis represents in all 3 graphs the mean ciliary body length (CBL). The 3 AL groups are depicted in colour on each graph (colour association is depicted on the vertical bar on the right)

The mean age was 42 years (SD ± 15.4, range 19–79 years). The mean body height was 173 cm (SD ± 8.9, range 148–200 cm) and the mean SE was − 1.64 diopters (SD ± 3.15, range − 14.5 to + 9 diopters). The mean AL was 24.19 mm (SD ± 1.65, range 19.8–32.2 mm) (Table 1). There was a significant correlation between SE and AL (*r*^2^ = 0.62, *p* < 0.0001) as well as between AL and body height (*r*^2^ = 0.02, *p* = 0.0002). Mean CBL correlated significantly with age (*r*^2^ = 0.11, *p* < 0.0001), AL (*r*^*2*^ = 0.23, *p* < 0.0001) and SE (*r*^2^ = 0.25, *p* < 0.0001) (Fig. 2). The mean CBL was 3351 μm (SD ± 459) with a range from 2184 to 4451 μm. In a subgroup of 40 patients, the mean distance between the limbus (Fig. 1, “L”) and the ciliary body (Fig. 1, “ACA”, which represents the starting point of our ciliary body length measurement) measured 795 μm. Comparing the CBL in the 4 quadrants, the superior-temporal quadrant showed the largest CBL with 3462 μm (SD ± 497.9), and the anatomically opposing inferonasal quadrant showed the smallest CBL with 3238 μm (SD ± 506). In the superonasal and inferotemporal quadrants, we measured very similar-sized CBLs with 3369 μm (SD ± 461) and 3314 μm (SD ± 540), respectively.

**Fig. 3:**
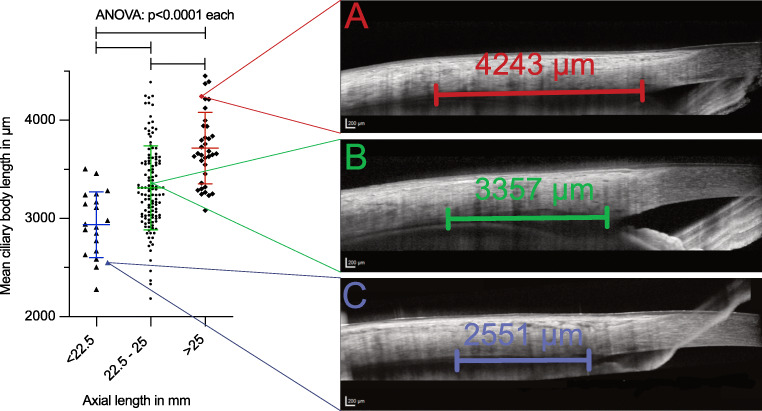
Graph showing the 3 groups defined by their axial length (short, normal and long) and the corresponding mean ciliary body lengths (CBL) of the individual eyes on the left with 3 anterior segment optical coherence tomography (ASOCT) slides on the right illustrating the CBL measurements. Each ASOCT image belongs to one sample of the 3 groups, whereby its exact position on the graph is indicated with thin lines. (A) ASOCT image of an eye in the long AL (> 25 mm) group with a rather long CBL. (B) ASOCT image of an eye in the moral AL (22.5–25 mm) group with a normal-sized CBL. (C) ASOCT image of an eye in the short AL (< 22.5 mm) group with a fairly short CBL

**Fig. 4:**
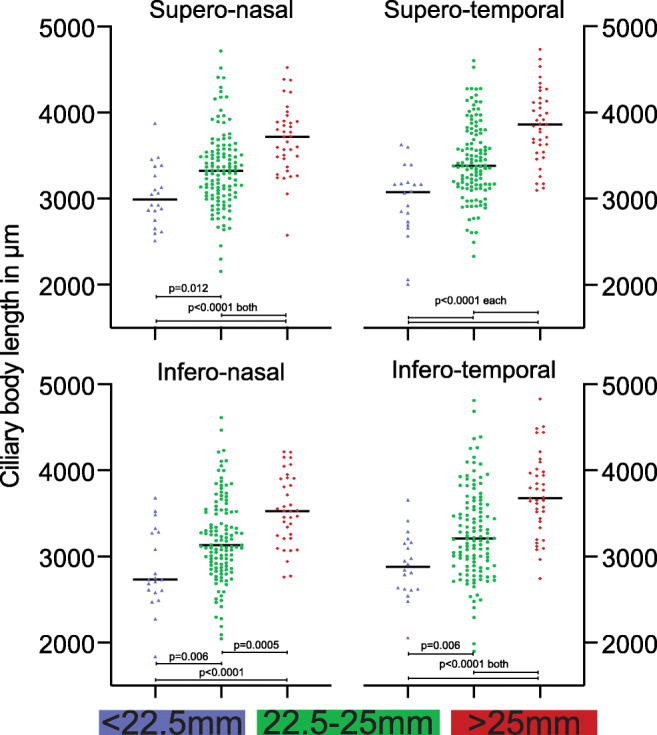
Comparison of the mean ciliary body length (CBL) in the 4 quadrants (superotemporal, inferotemporal, inferonasal and superonasal) between the 3 groups, which are defined by their axial length (AL). Each graph depicts the distribution of the mean CBL in the respective quadrant between the 3 AL groups. Below the graphs, a colour-coded bar depicts the colour association to the 3 AL groups

Three separate groups were defined by their AL with a normal AL group (AL ranging from 22.5 to 25 mm, *n* = 123), a short AL group (AL < 22.5 mm, *n* = 20) and a long AL group (AL > 25 mm, *n* = 36). We found a mean CBL in the normal AL group of 3311 μm (SD ± 427), in the short AL group of 2936 μm (SD ± 335) and in the long AL group of 3715 μm (SD ± 365). After verifying a normal distribution using the d’Agostino and Pearson omnibus K2 normality test, we analysed and compared the 3 groups using a one-way ANOVA test. The mean CBL showed significant differences between the 3 groups (*p* < 0.0001, Fig. 3). (For an overview of the groups, see Table 2.) In a subanalysis concerning the 4 quadrants (ST, SN, IT and IN), we also found significant differences between the 3 groups for each quadrant’s CBL (Fig. 4).

**Table 2:** Illustration of the 3 groups defined by axial length (*AL*) with mean values and standard deviations (*SD*). *SE*, spherical equivalent; *CBL*, ciliary body length; *n*, number of subjects included in group

	AL mean ± SD (mm)	SE mean ± SD (dpt)	CBL mean ± SD (μm)
Short AL group (< 22.5 mm, *n* = 20)	21.96 ± 0.58	1.54 ± 2.29	2936 ± 334.9
Normal AL group (22.5–25 mm, *n* = 123)	23.77 ± 0.66	− 0.86 ± 1.7	3311 ± 427.3
Long AL group (> 25 mm, *n* = 36)	26.53 ± 1.77	− 5.51 ± 3.75	3715 ± 364.8

## Discussion

A significant correlation between the mean CBL and AL was found. This finding suggests that the ciliary body grows or stretches as does the sclera in case of globe elongation in myopia. This is an interesting finding, as the size of the ciliary body dictates the position of the pars plana and thus the anatomic site of surgical entrance for posterior segment surgery and intravitreal injections. A distance posterior to the limbus of 3.5 to 4.0 mm is considered a safe distance to be sure to pierce the pars plana and not damage the retina or the lens [14]. However, in our cohort, a range of 2170 μm or 2.2 mm between the shortest and longest CBL and a difference of 770 μm or 0.77 mm in mean CBL between the group with short AL and the group with long AL are demonstrated. To put things in perspective, this range of 0.77 mm exceeds the variation of 0.5 mm between the lower and upper limits of the advised safety distance from the limbus (3.5–4.0 mm). This leads to the conclusion that the CBL should be taken into account for a safe pars plana access. The AL seems to represent an easy and quickly measurable surrogate marker of the CBL in light of the significant correlation with CBL in our sample. We would argue that in case of short eyes (AL < 22.5 mm), one should err on the short side of the recommended distance (3.5 mm) and in a long eye (AL > 25 mm) on the long side (4.0 mm). A limitation of this approach is the variability of the injection site in daily clinics. As the distance from the limbus is measured by hand with an appropriate instrument and marked by impression or by a pen by the operating surgeon, there will be some variability in the distance, especially in the face of the small room for error when marking distances in fraction of millimetres. This variability of the injection site can be depicted by infrared imaging, whereby the injection points can be easily defined and show a marked spread [15]. Therefore, it will be difficult to put these miniscule changes of distance into practice.

A further limitation of our approach is that in our sample size of 200 eyes, we only had a few outliers (*n* = 20 for the short AL group and *n* = 36 for the long AL group) reaching or surpassing the aforementioned cut-offs. The reported information about CBL varies greatly in the literature. Data from autopsy reports range from 4.5 to 6.3 mm [16–18]. A study measuring the CBL using UBM found a mean ciliary body length of 5.43 mm (SD ± 0.58, range 4.0–6.4 mm) [12]. In comparison, our results came in significantly shorter (mean 3351 μm, range 2184–4451). There are various possible explanations for these shorter measurements. One would be false measurements. As measurements were conducted by 2 independent examiners and the individual measurements in 3 different B-scans reached an ICC of 0.91, we deemed this possibility unlikely. One explanation could be varying definitions regarding anatomic landmarks. We measured from the iridocorneal angle to the last discernible mass of the pars plana (see Figs. 1 and 3). However, it is possible that we measured only the pars plicata and omitted inclusion of the pars plana because of reasons of visibility in the OCT image. In this context, it should be mentioned that the iridocorneal angle lies more posteriorly than the external surgical limbus, which is used as landmark to determine the correct intravitreal injection site. In a subgroup of 40 patients, we measured this distance to be 795 μm in our cohort. A third possibility is a different imaging technique used across these studies. Previously published literature used fixated tissue [16–18] or UBM [12], whereby the measurements started from the scleral spur and not the iridocorneal angle and additionally have taken the curvature of the eye into account. Another possibility are measurement artefacts due to these more invasive or manipulative techniques. Through fixation of tissue in formalin, shrinkage may be induced [19]. In UBM, slight touching, and thereby impression of the sclera, is necessary which may have implications on measured length. In theory, ASOCT provides the most unbiased in vivo examination technique. However, in this case, the differences are more likely to originate from the different landmarks used for the measurements as highlighted previously. Despite the differences in total CBL, the data is fairly consistent and is in keeping with previous reports showing the greatest extent of the CBL in the superior quadrant. Another limitation is that this data is only valid for full-grown eyeballs as only adults > 18 years have been included. For children, further data regarding the relation of the CBL and AL has to be gathered to draw conclusions. Intravitreal injections are nowadays a very common procedure; and though complications are rare, they are potentially vision threatening. Complications, which might occur due to entry in the surrounding areas of the pars plana, are for example rhegmatogenous retinal detachments (RRD) and vitreous haemorrhages (VH) [20, 21]. Both adverse events can lead to a drastic decline in vision.

## Conclusion

Our findings suggest that the axial length should be taken into account when choosing the exact distance to the limbus for pars plana access in intravitreal injections and vitreoretinal surgery to further reduce the risk of complications. A larger number of patients is needed to give a definite recommendation regarding an axial length–based injection site, but for now these findings suggest that it would be advisable to prefer the shorter or longer recommended distance posterior to the limbus (3.5 or 4.0 mm), which correlates with the respective axial length. On the basis of our data in case of an axial length of > 25 mm, a distance from the limbus of 4.0 mm should be chosen and in case of an AL of < 22.5 mm, a distance of 3.5 mm from the limbus seems adequate.

## Data Availability

The data that support the findings of this study are available on request from the corresponding author. The data are not publicly available due to them containing information that could compromise research participant privacy.
